# An Aberrant Right Subclavian Artery Causing Severe Esophageal Compression: A Case Report

**DOI:** 10.7759/cureus.79527

**Published:** 2025-02-23

**Authors:** Nektarios Galanis, Dimosthenis Chrysikos, Michail Palaios, Ameer Shehade, Panagiotis Georgakopoulos, Dimitrios Patsouras, Theodore Troupis

**Affiliations:** 1 Anatomy, National and Kapodistrian University of Athens School of Medicine, Athens, GRC

**Keywords:** aberrant right subclavian artery, dysphagia lusoria, esophageal compression, kommerell’s diverticulum, vascular anomalies

## Abstract

An aberrant right subclavian artery (ARSA), a congenital vascular anomaly, can cause significant esophageal compression, leading to a condition known as dysphagia lusoria (DL). We present the case of a 44-year-old man with progressively worsening dysphagia and odynophagia over the last six months, resulting in severe weight loss and dietary restrictions. Imaging techniques revealed esophageal stenosis caused by external compression from an ARSA arising from the posterior wall of the distal aortic arch, accompanied by a Kommerell’s diverticulum. Computed tomography angiography confirmed the aberrant origin, retroesophageal course, and vascular anomaly. Although surgical intervention involving ligation and excision of the retroesophageal artery segment with a right carotid-subclavian bypass was recommended, the patient opted for conservative management. This case highlights the importance of advanced imaging techniques in diagnosing DL and guiding treatment decisions. Regular follow-up remains essential to monitor disease progression and manage potential complications.

## Introduction

The subclavian artery is a paired arterial vessel located just inferior to the clavicles and is one of the largest arteries of the thorax. Typically, the right and left subclavian arteries have distinct origins. The left subclavian artery arises directly from the aortic arch, distal to the origin of the left common carotid artery, marking the beginning of the descending aorta. The right subclavian artery and the right common carotid artery originate from the brachiocephalic trunk. Despite their different origins, the two subclavian arteries normally follow a similar course in the neck region. They travel toward the axillary region from their origins, passing posterior to the anterior scalene muscles and anterior to the middle scalene muscles. Based on their relationship to the scalene muscles, the subclavian arteries are divided into prescalene, retroscalene, and postscalene segments. Beyond the lateral border of the first rib, the subclavian artery becomes the axillary artery. The subclavian artery supplies blood to the upper limbs, thorax, neck, and brain [1-3].

We present a rare case of an aberrant right subclavian artery (ARSA) arising from the posterior wall of the distal part of a left-sided aortic arch, as a fourth branch. Its retroesophageal course caused significant compression, resulting in severe dysphagia and weight loss. Informed consent was obtained from the patient to present this anonymized case.

## Case presentation

A 44-year-old man presented to the otolaryngology outpatient clinic with severe, progressively worsening dysphagia and odynophagia over the past six months. During this time, he lost 10 kilograms and had been restricted to consuming mostly liquid foods for three to four weeks. He reported extreme weakness and a marked decline in quality of life. His medical history revealed chronic dysphagia that had been manageable until approximately six months earlier.

**Figure 1:**
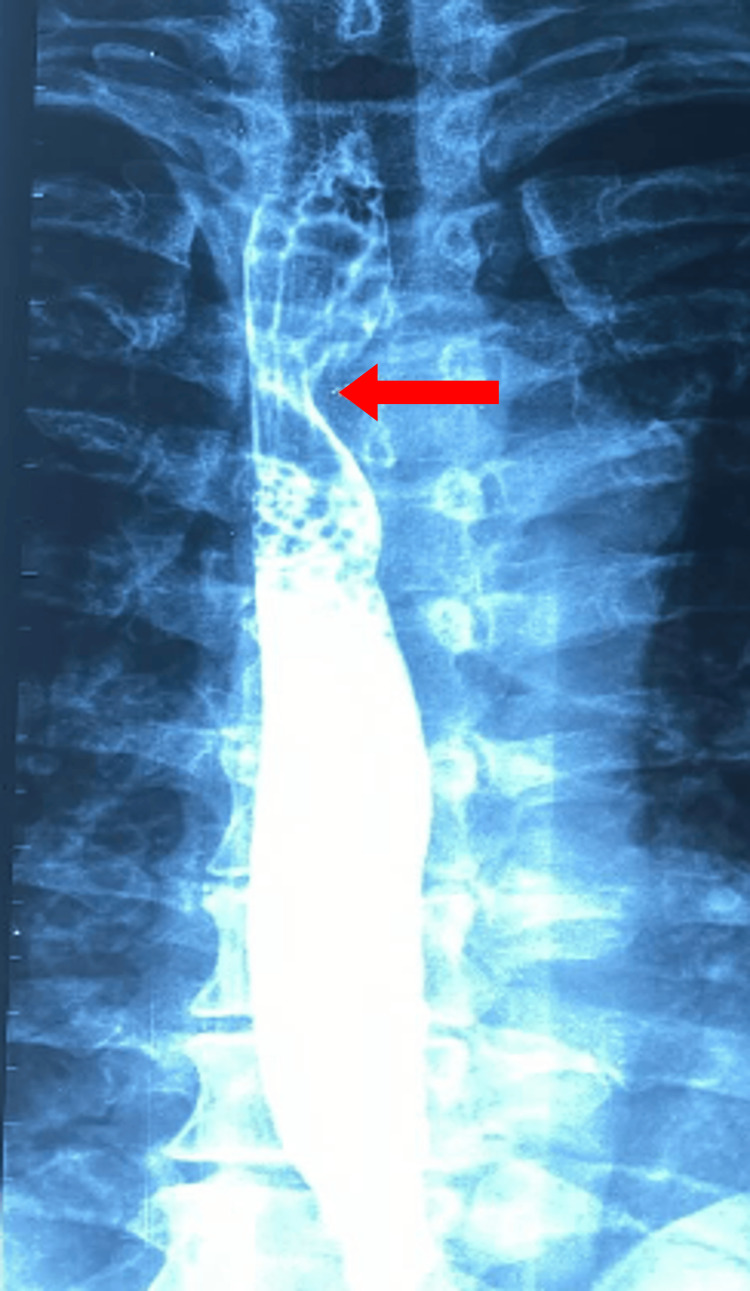
Esophagography demonstrating esophageal stenosis (red arrow).

Physical examination by the otolaryngologist revealed no abnormalities. The patient was referred to a gastroenterologist for further evaluation. Gastroscopy showed no lesions of the esophageal mucosa but identified significant stenosis 24 to 26 cm from the incisors, likely due to external compression. Esophagography confirmed this suspicion (Figure 1).

**Figure 2:**
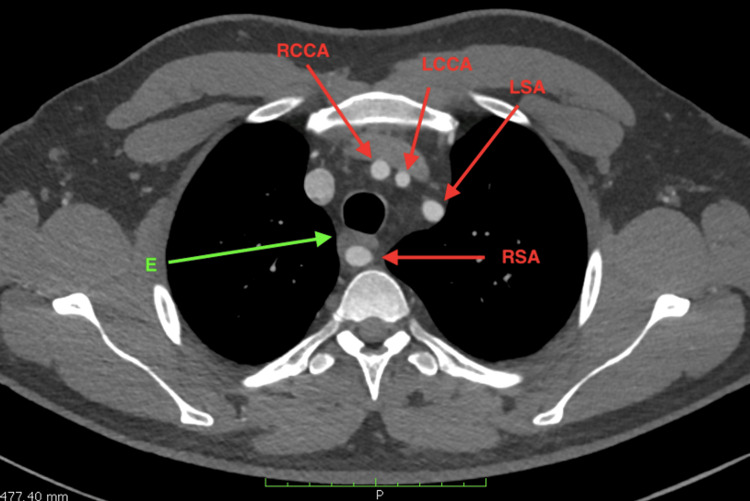
CT angiography: horizontal section at the level of the third thoracic vertebra. The RCCA, LCCA, and LSA are identified. The RSA is seen ventral to the third vertebral body, causing significant compression of the esophagus. CT: computed tomography; RCCA, right common carotid artery; LCCA, left common carotid artery; E: esophagus; LSA: left subclavian artery; RSA: right subclavian artery.

**Figure 3:**
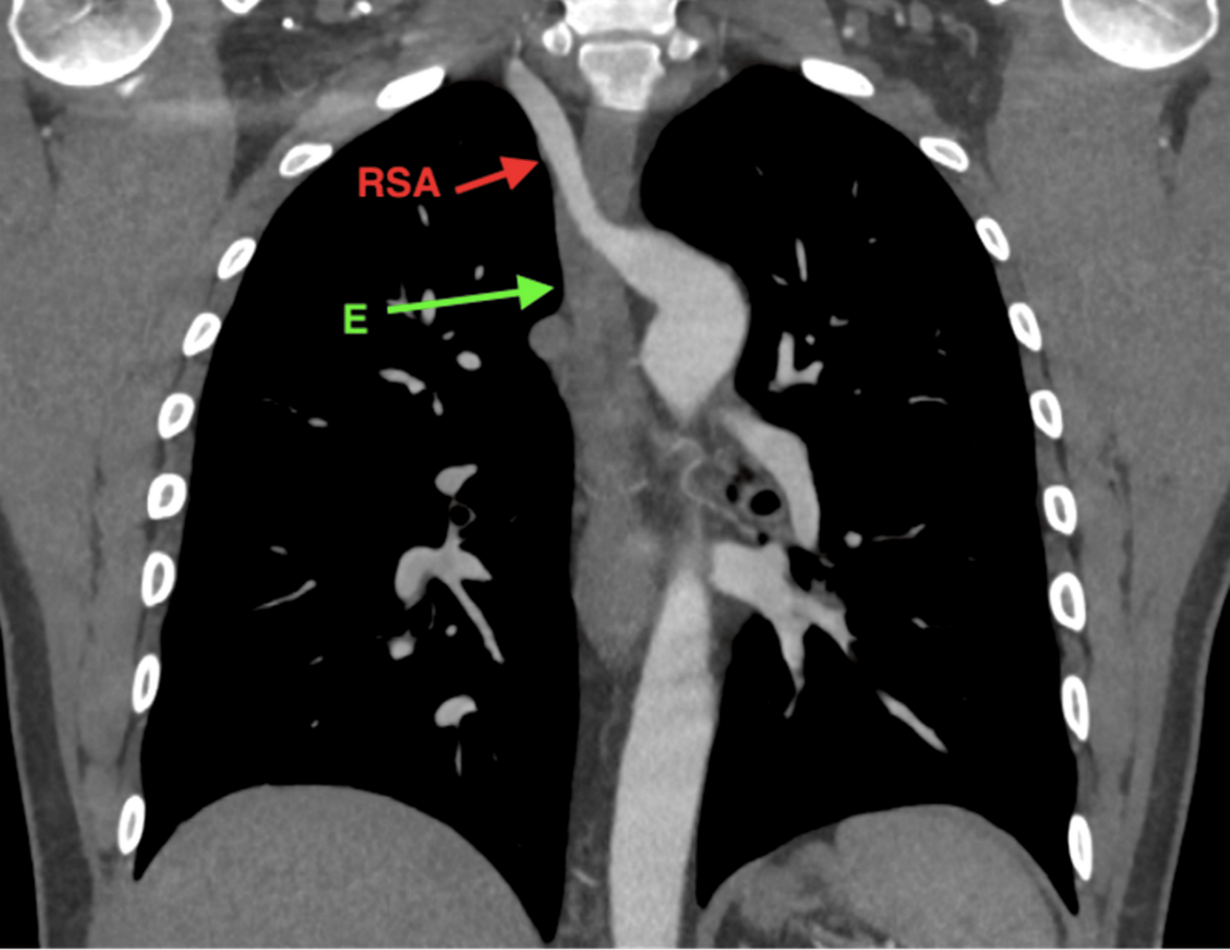
CT angiography: coronal section of the thorax at the level of the esophagus. The ascending course of the RSA from the left to the right is visualized, originating from the posterior wall of the distal aortic arch. Note the slight enlargement at the origin of the RSA. CT: computed tomography; E: esophagus; RSA, right subclavian artery.

**Figure 4:**
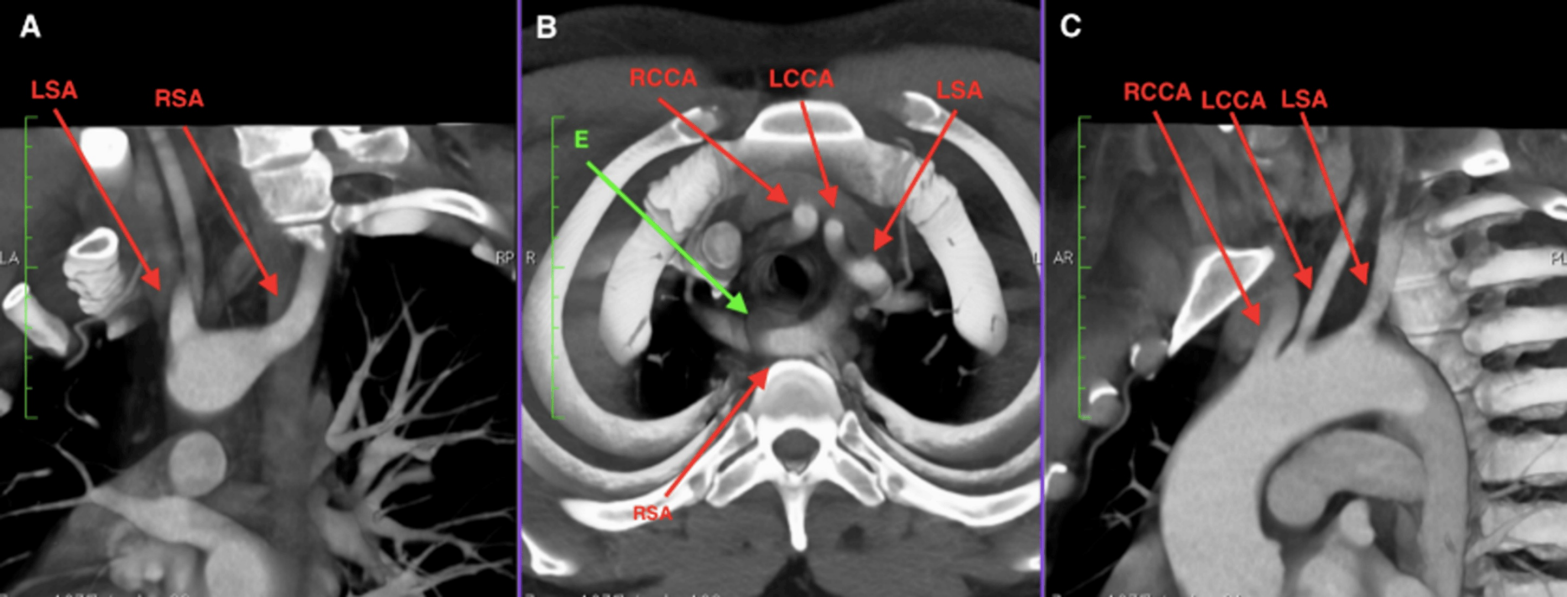
Computed tomography (CT) angiography of the upper thorax with 30-mm thick slabs: (A) Coronal section rotated 45º to the right of the vertical axis. (B) Horizontal section at the level of the third thoracic vertebra. (C) Coronal section rotated 55º to the left of the vertical axis. CT: computed tomography; LSA: left subclavian artery; RSA: right subclavian artery; E: esophagus; RCCA: right common carotid artery; LCCA: left common carotid artery.

Computed tomography angiography (CTA) of the thorax revealed an abnormal origin of the right common carotid artery in the anatomical position of the brachiocephalic artery. The right subclavian artery originated aberrantly from the posterior surface of the distal aortic arch as a fourth branch. It traveled pre-vertebrally at the level of the third and fourth thoracic vertebrae and passed posterior to the esophagus, exerting significant pressure on it. The artery then ascended to the right, and a slight enlargement of its origin was noted (Figures 2-4).

Given the patient’s young age and the life-threatening esophageal compression leading to malnutrition and rapid weight loss, surgical intervention was recommended. The proposed procedure involved ligation and excision of the retroesophageal segment of the right subclavian artery via a left thoracotomy, followed by a right carotid-subclavian bypass. After a detailed discussion of the risks and benefits of surgery, the patient opted for conservative management. This method includes dietary modification and instructions to chew well and eat slower. This is an effective management strategy, provided that patients can maintain their weight and good nutritional status, such as in our case. Surgical intervention or a minimally invasive approach with stent becomes necessary for patients who show no improvement with these dietary and eating modifications and those who are not amenable to these conservative management techniques. Regular follow-up evaluations have been planned.

## Discussion

In this case, the ARSA, the most common aortic arch anomaly (prevalence: 0.3%-1%) [4], arose from the posterior wall of the distal aortic arch, exerting external pressure on the esophagus and causing dysphagia. This specific presentation is described in the literature as dysphagia lusoria (DL), which is esophageal or tracheal compression caused by congenital malformations of the aortic arch [5].

Embryologically, ARSA results from the abnormal interruption of the fourth aortic arch during development, leading to its persistence rather than regression [6]. Bayford first described symptomatic esophageal compression due to ARSA in 1794, coining the term “dysphagia lusoria,” derived from the Latin phrase lusus naturae (jest of nature) [7]. Dysphagia is defined as difficulty swallowing, and in DL, patients often report a sensation of food sticking in the pharynx and pain during swallowing (odynophagia) [8], as observed in our patient. Additional consequences include weight loss, prolonged mealtimes due to excessive chewing, and avoidance of previously consumed solid foods [8].

Aneurysms at the origin of the aberrant subclavian artery are present in a small percentage of cases [9]. This condition, visible in our patient (Figures 3, 4), is known as Kommerell’s diverticulum (KD) [9-11]. KD is a rare congenital anomaly characterized by a remnant of the fourth dorsal aortic arch that forms a focal vascular sac at the origin of the ARSA [4]. KD is frequently associated with symptomatic cases of ARSA [12]. Histopathologic studies of KD have shown cystic medial necrosis in the diverticulum wall, which is associated with an increased risk of aortic dissection and rupture [11]. A KD diameter of ≥3 cm from the opposite aortic wall to the tip of the diverticulum is the most accepted threshold for prophylactic intervention [13].

Various methods can diagnose DL, including barium esophagography [14], endoscopy, computed tomography, magnetic resonance angiography, and digital subtraction angiography [5]. Multi-detector CTA is the preferred method because it provides detailed visualization of the vasculature [5]. In our patient, esophagography demonstrated stenosis caused by external compression from the ARSA (Figure 1). Subsequently, CTA confirmed the aberrant origin and retroesophageal course of the ARSA and the presence of KD.

The ideal treatment for DL depends on the clinical presentation, including the severity of dysphagia, weight loss, and patient age [14]. In mild cases, management involves dietary modifications, increased chewing, and extended mealtime duration, accompanied by close follow-up [15]. Surgical intervention is warranted for persistent symptoms, vascular rings, KD, or dissecting aneurysms [15]. Surgical treatment aims to restore healthy blood flow to the right arm [6]. It commonly involves anastomosis of the divided subclavian artery to the right common carotid artery (right carotid-subclavian bypass) [16] or directly to the aortic arch using a prosthetic graft [6]. Various surgical approaches have been described, including right and left thoracotomies, cervical incisions, median sternotomies, and combinations of these techniques [6]. In cases of KD, additional intervention beyond the division of the ARSA is recommended due to the risk of aneurysmal rupture and dissection [9].

In this case, we recommended a surgical approach involving ligation and excision of the retroesophageal segment of the ARSA via left thoracotomy, followed by a right carotid-subclavian bypass. However, after a detailed discussion of the risks, benefits, and potential complications, the patient opted for conservative management with regular follow-up examinations.

## Conclusions

We describe a rare case of DL caused by an ARSA, leading to esophageal compression, severe weight loss, and avoidance of solid foods. Imaging modalities, particularly esophagography, and CTA, were instrumental in visualizing the origin and course of the ARSA and diagnosing KD. Although surgical treatment was recommended, the patient elected to pursue conservative management after being informed of the potential risks and benefits. This case highlights the importance of considering vascular anomalies such as ARSA in the differential diagnosis of unexplained dysphagia, particularly when standard evaluations fail to identify an intraluminal cause. Early recognition and imaging are critical to ensure timely diagnosis and appropriate management, which may involve a multidisciplinary approach to balance the risks and benefits of surgical versus conservative treatment.

## References

[1] Standring S (2016). Gray's anatomy. https://books.google.gr/books?hl=el&lr=&id=qlgAEAAAQBAJ&oi=fnd&pg=PP1&dq=1.+Standring,+S.+(2016).+Gray%27s+Anatomy+(41st+ed.).+Edinburgh:+Elsevier+Churchill+Livingstone&ots=snQdaOwpCo&sig=VprLhoAsNySvDMkah-KJR4x0utw&redir_esc=y#v=onepage&q&f=false.

[2] Netter F (2019). Atlas of human anatomy. https://cmc.marmot.org/Record/.b27087888.

[3] Moore KL, Dalley AF, Agur AMR (2014). Clinically oriented anatomy. https://books.google.gr/books?id=-Le5bc5F0sYC&printsec=frontcover&hl=el#v=onepage&q&f=false.

[4] Gnanapandithan K, Rahni DO, Habr F (2014). Intermittent esophageal dysphagia: an intriguing diagnosis. Dysphagia lusoria. Gastroenterology.

[5] Alper F, Akgun M, Kantarci M (2006). Demonstration of vascular abnormalities compressing esophagus by MDCT: special focus on dysphagia lusoria. Eur J Radiol.

[6] Whitley A (2001). Dysphagia lusoria: a case study. J Vasc Nurs.

[7] Bayford D (1794). Account of a single case of obstructed deglutition. Mem Med Soc London.

[8] Erami C, Charaf-Eddine A, Aggarwal A, Rivard AL, Giles HW, Nowicki MJ (2013). Dysphagia lusoria in an infant. J Pediatr.

[9] Dieffenbach BV, Sharma G, Shah SK, Menard MT, Belkin M (2020). Aberrant subclavian artery division and revascularization by a supraclavicular approach for definitive or staged treatment of dysphagia lusoria. J Vasc Surg.

[10] Keum B, Kim YS, Jeen YT (2006). Dysphagia lusoria assessed by 3-dimensional CT. Gastrointest Endosc.

[11] Tsai MC, Lee CT, Wang WL (2021). An unusual cause of dysphagia. Gastroenterology.

[12] Feezor RJ, Lee WA (2007). Dysphagia lusoria. J Vasc Surg.

[13] Cho JS (2022). Unraveling the enigma of an aberrant subclavian artery (arteria lusoria). J Vasc Surg.

[14] De Caluwe E, Verhaegen S, Van Roey G, Janssens J, Van Gool S (2012). Dysphagia lusoria caused by a right-sided aorta. Acta Gastroenterol Belg.

[15] Kang MS (2014). Dysphagia lusoria caused by Kommerell's diverticulum of the aberrant left subclavian artery. Clin Gastroenterol Hepatol.

[16] Mittal SO, Jabbari B, Machado DG (2012). A common symptom in two uncommon coexistent conditions: glomus jugulare tumor and dysphagia lusoria. Clin Neurol Neurosurg.

